# Spatial–Temporal Analysis of Air Pollution, Climate Change, and Total Mortality in 120 Cities of China, 2012–2013

**DOI:** 10.3389/fpubh.2016.00143

**Published:** 2016-07-18

**Authors:** Longjian Liu, Xuan Yang, Hui Liu, Mingquan Wang, Seth Welles, Shannon Márquez, Arthur Frank, Charles N. Haas

**Affiliations:** ^1^Department of Epidemiology and Biostatistics, Drexel University Dornsife School of Public Health, Philadelphia, PA, USA; ^2^Shanghai Advanced Research Institute, Chinese Academy of Sciences, Shanghai, China; ^3^Department of Environmental and Occupational Health, Drexel University Dornsife School of Public Health, Philadelphia, PA, USA; ^4^Department of Civil, Architectural, and Environmental Engineering, Drexel University College of Engineering, Philadelphia, PA, USA

**Keywords:** air pollution, climate change, healthy city, China

## Abstract

China has had a rapid increase in its economy over the past three decades. However, the economic boom came at a certain cost of depleting air quality. In the study, we aimed to examine the burden of air pollution and its association with climatic factors and health outcomes using data from Chinese national and city-level air quality and public health surveillance systems. City-level daily air pollution index (API, a sum weighted index of SO_2_, NO_2_, PM_10_, CO, and Ozone) in 120 cities in 2012 and 2013, and its association with climate factors were analyzed using multiple linear regression analysis, spatial autocorrelation analysis, and panel fixed models. City-level ecological association between annual average API and total mortality were examined using univariate and partial correlation analysis. Sensitivity analysis was conducted by taking the consideration of time-lag effect between exposures and outcomes. The results show that among the 120 cities, annual average API significantly increased from 2012 to 2013 (65.05 vs. 75.99, *p* < 0.0001). The highest average API was in winter, and the lowest in summer. A significantly spatial clustering of elevated API was observed, with the highest API in northwest China in 2012 and with the highest in east China in 2013. In 2012, 5 (4%) of the 120 cities had ≥60 days with API >100 (defined as “slightly polluted”), however, it increased to 21 cities (18%) that experienced API >100 for ≥60 days in 2013. Furthermore, 16 cities (13%) in 2012 and 35 (29%) in 2013 experienced a maximum API >300 (defined as “severely polluted”). API was negatively and significantly correlated with heat index, precipitation, and sunshine hours, but positively with air pressure. Cities with higher API concentrations had significantly higher total mortality rates than those with lower API. About a 4–7% of the variation in total mortality could be explained by the difference in API across the nation. In conclusion, the study highlights an increased trend of air pollution from 2012 to 2013 in China. The magnitude of air pollution varied by seasons and regions and correlated with climatic factors and total mortality across the country.

## Introduction

Urban air pollution is of increasing global concern as it has great impacts on the environment and public health. China has had a rapid increase in its economy over the past three decades; however, the economic boom also came at a certain cost of depleting air quality. Outdoor air pollution has become a top environmental concern in China (1–4). In addition, as the use of motor vehicles has been increasing, air pollutants have shifted from those arising from conventional coal combustion to more emissions from mixed sources (2). In urban areas, emissions from residential energy combustion, transportation, and industry are the main sources of air pollution; while in rural areas, pollution most commonly originates from combustion and solid fuel (5). The burden of air pollution in China has been examined by some studies (6–10). For example, the effect of air pollutants (PM_10_, ozone, nitrogen dioxide, and sulfur dioxide) per 10-μg/m^3^ increases in concentration was found to be significantly correlated with risk of cardiovascular and respiratory morality in three Chinese cities (Hong Kong, Shanghai, and Wuhan) (6), a significant association between air pollution and respiratory mortality in the elderly was observed in a study of 32 Chinese cities by Zhou and colleagues (7), and results from China Air Pollution and Health Effects Study showed that a 10-μg/m^3^ increase in 2-day moving-average PM_10_ was associated with a 0.35% increase of total mortality, 0.44% increase of cardiovascular mortality, and 0.56% increase of respiratory mortality. Females, elderly, and residents with low educational attainment appeared to be more vulnerable to PM_10_ exposure (9). Findings from these studies highlighted the risk of exposure to air pollutants and human health. However, these studies had limitations of their representativeness due to a small sample size, and data from few selected cities. Furthermore, although it is known that mortality from cardiovascular disease and respiratory diseases significantly increase in winter as compared with the other seasons, limited information is available on the association between climate change and air quality, and their effect on health outcomes. A better understanding of this complex association will help health professionals and policy makers to have a better prevention strategy and approach for air quality control and public health improvement. In this study, we aimed to use large-scale data, with a better representativeness from multiple sources on air quality measures, climate factors, and health outcomes that are collected using standard study design and survey approach, and reported by the Ministry of Environmental Protection of the People’s Republic of China, China Meteorological Data Sharing Service System, and National Health Statistics Center of the People’s Republic of China (11–14). In the study, we had two specific aims (1) to examine the magnitude of air pollution by regions and spatial variation and its association with climatic factors and (2) to examine the association between air quality and health outcomes (assessed by total mortality) among major cities of the country. Findings from the study will not only provide update evidence on the burden of air pollution but also will add new evidence to the body of the literature on the association between air pollution and health outcomes using data from national environmental and health care surveillance systems. Given the data use from the national reports, findings from the study may serve as an important base for further studies.

## Materials and Methods

### Air Pollution Data

In China, daily air pollution data are reported through China National Urban Air Quality publishing platform, endorsed by the Ministry of Environmental Protection of the People’s Republic of China (MEP) (11). Air pollution index (API), a sum weighted air pollution indicator, was reported daily for 120 major cities in 2012 and 2013 by the MEP (11). The overall goal of the air quality report by the MEP is to provide timely information to the public, and help citizens understand how local air quality changes over time. The API is calculated from daily measures of five major atmospheric pollutants: particulate matters, SO_2_, NO_2_, CO, and Ozone. The average daily concentrations of pollutants in each city were computed using a centering method and the total API for each city was calculated using liner interpolation approach (1, 15, 16). The mathematical formula is specified as follows:

(1) $${\text{API}}_{\text{i}}={\text{ (API}}_{\text{U}}-{\text{API}}_{\text{L}}{\text{)/(C}}_{\text{U}}-\;{\text{C}}_{\text{L}}\text{)}*\left({\text{C}}_{\text{i}}\;-\;{\text{ C}}_{\text{L}}\right)\;+\;{\text{API}}_{\text{L}}$$

(2) $$\text{API}=\text{max}\left({\text{API}}_{\text{i}}\right)$$

where API_i_ is the index value for individual pollutant i. A daily index value is calculated for each air pollutant. C_i_ is the observed concentration of pollutant i. C_U_ and CL are the upper and lower limits of the interval, within which lies the C_i_. API_U_ and API_L_ are the upper and lower limits of the corresponding API interval. The API is defined as the maximum of API_i_ and the pollutant responsible for the highest index value is the “main pollutant.”

It should be noted that air quality data are formally managed and reported by MEP in China. Data for individual pollutant reports are not available to the public and researchers. API, a sum weighted index, calculated using an international standard approach (16), is the only report and available on air quality information in and before 2013 (7, 11, 15, 16). Therefore, we examined the associations between air quality, climate factors, and health outcomes using API as an air quality indicator only.

### Meteorology Data

Daily meteorology data were collected from 194 meteorological observation stations in China, which is available from the Chinese Meteorological Data Sharing Service System (12, 17). In the analysis, we included five major meteorological indicators: temperature, relative humidity, precipitation, sunshine hours, and pressure. Heat index (HI) was further estimated using the regression equation proposed by Rothfusz (18).

$$\begin{array}{l} \text{HI =}-\text{42.379 + 2.04901523*T +10.14333127*RH}-\text{0.22475541*T*RH}-\text{0.00683783*T*T }-\text{0.05481717*RH*RH +}\\ \text{ 0.00122874*T*T*RH + 0.00085282*T*RH*RH}-\text{0.00000199*T*T*RH*RH} \end{array}$$

Where T is temperature in degrees Fahrenheit and RH is relative humidity in percent.

### Health Outcomes Data

City-level annual total mortality rates in 2010 and 2013 were collected from Chinese Annual Health Statistics, and Statistical Communique of the People’s Republic of China on National Economic and Social Development (13, 14).

### Covariates

City-level population size and gross domestic product (GDP), GDP per capita, and percentage of residents aged 60 years and older were acquired from China Statistical Yearbooks and city-level health statistics (13, 19–37).

### Statistical Analysis

In the first group analysis, univariate analyses were conducted to describe the patterns of daily, weekly, seasonal, and yearly average of API across cities and regions. Spline regression technique was also applied to depict API variation by time. We classified seasons as spring (March, April, and May), summer (June, July, and August), fall (September, October, and November), and winter (December, January, and February). Meanwhile, because this classification may not be very accurate to distinguish seasonal effects on API due to a large land area of China (38), we examined and presented the patterns of API by temperatures and weeks using three-dimensional technology. To examine regional difference, we classified six regions using a standard method that is commonly applied in China (39). The six regions are as follows: North China (15 cities, including Beijing, Tianjin, and cities from Hebei, Shanxi, and Inner Mongolia provinces), Northeast China (12 cities from Liaoning, Jilin, and Heilongjiang provinces), East China (35 cities, including Shanghai, and cities from Jiangsu, Zhejiang, Anhui, Fujian, Jiangxi, and Shandong provinces), South China (30 cities from Henan, Hubei, Hunan, Guangdong, Guangxi, and Hainan provinces), Southwest China (15 cities, including Chongqing, and cities from Sichuan, Guizhou, Yunnan, and Tibet provinces), and Northwest China (13 cities from Shanxi, Gansu, Qinghai, Ningxia, and Xinjiang provinces).

In the second group analysis, spatial patterns of mean API by geographic and three time periods (years) were described using Arc Geographic Information System – Ring map technology (GIS Version 12, Redlands, CA, USA) (40). In the analysis, 120 cities had API measures in both 2012 and 2013, and 80 cities had API measures in 2007–2010, 2012, and 2013. Because the number of cities with API measures were limited by years before 2013, we calculated the annual average API of 2007–2010 (i.e., a city that had at least two annual API measures during 2007–2010 is included in the analysis) in order to increase the number of cities that can be used to test an overall trend of API from 2007–2010 to 2013. It should be also noticed that only 49 matching cities that had both measures of API and climate factors in 2012 and 2013. Therefore, data analysis for the association between API and climate factors was conducted using data from the 49 cities.

In the third group analysis, because of the nature of the spatial-based cross-sectional and time-series datasets, we applied Panel analysis methods to take into consideration the temporal heterogeneities in API and climate factors. Panel analysis is a statistical method, widely used in environmental science, social science, epidemiology, and economics, which deals with two and “*n*”-dimensional (in and by the – cross-sectional/times series time) panel data (7, 41, 42). Data are usually collected for same individuals over time and then a regression is run over these dimensions. There are three commonly used Panel analysis approaches (1) independently pooled model, it assumes that there are no unique attributes of individuals within the measurement set (e.g., spatial), and no universal effects across time; (2) fixed effect model, it assumes that there are unique attributes of individuals that are not the results of random variation (location-specific effect) and that do not vary across time (time-specific effect); and (3) random effect model, it assumes that there are unique, time constant attributes of individuals that are the results of random variation and do not correlate with the individual regressor (7, 41–43). In the study, we tested the random effect’s hypothesis (i.e., the null hypothesis of no correlation among effects and regressors) using Hausman test (43), the result showed the time-specific effects of climate factors (HI, precipitation, sunshine, and pressure) on API were not randomly distributed (*p* < 0.001), and the intraclass correlation coefficients of the regressors were highly significant, except for a non-significant correlation between precipitation and pressure in 2013 (*p* = 0.188) (data not shown). Therefore, we only reported the results from Panel analysis fixed effect models.

In the fourth group analysis, the association between city-level annual average API and annual total mortality rates were examined using univariate and partial (adjusted) correlation analysis. In the partial correlation analysis, we adjusted for city-level socioeconomic status (assessed by GDP per capita) and percentage of residents who were aged 60 and older in individual cities. In the final report, we highlighted the results of adjustment for each covariate individually, because of giving a relatively small sample size and applying a city-level ecological study design, increasing the number of covariates in the adjustment would lead to significantly lower statistical powers.

Finally, by considering a possible time-lag effect between exposure and health outcomes, we conducted a set of sensitivity analyses to examine the association between annual average air quality measured in early years (2002–2010) and total mortality in late years (2012 and 2013) (44). The analysis included (1) testing the association between air quality in 2003 and 2004 and total mortality in 2012 and 2013 (i.e., assuming a 10-year time-lag effect between exposure and total mortality), (2) testing the association between air quality in 2008 and 2009 and total mortality in 2012 and 2013 (i.e., a 5-year time-lag effect between exposure and total mortality), and (3) repeating the analysis by applying a 4-year annual average estimate of air quality in 2002–2005, and in 2007–2010 to test the associations between air quality and total mortality (i.e., assuming a 5- and 10-year time-lag effect). This method by calculating annual average values of air quality measured in multiple years allowed us to increase the sample size, because some cities they had at least 2 years measures during this time period can be included. Furthermore, we assumed that a 4-year (or at least 2-year) average estimate was more reliable than that would be found for a single year (44, 45). The reason for the test of a 5- and 10-year time-lag effect is on the basis of previous observational studies (46–49). The biological rationale is that chronic disease develops over a long period of time, typically for years, during which distinct biological events need to take place, and that the relevant etiologic agents may have an impact on one or more “steps” in this process. It is, therefore, important to account for an adequate interval of time between exposure and outcome (49). For example, in Pope and colleagues report, an association was studied between mortality data for 1982–1998 and air pollution measures for 1999–2000 (50). An association between zinc component air releases for 1991–2000 using the U.S. Toxics Release Inventory database and cardiovascular mortality for 2006–2010 was examined in Chen and colleagues’ study (46). In our study, we tested whether a 5-year and/or 10-year time-lag effects of API on total mortality exist on the basis of the available datasets.

All statistical analyses were conducted using SAS software, version 9.3 (SAS Institute, Cary, NC, USA) (41). The level of significance was set at a two-sided test at *p* ≤ 0.05.

## Results

### Characteristics of the Study Sample

Table 1 shows that among the 120 cities, daily mean API was significantly higher in 2013 than in 2012 (75.99 vs. 65.05, *p* < 0.0001). The average temperature in 193 sample cities was 10.84°C in 2012, and 11.55°C in 2013 (*p* < 0.0001). Similar to these, lower average HI and sunshine hours were observed in 2012 than in 2013, except a higher precipitation was observed in 2012 than in 2013. There were no significant differences in the total and per capita GDP (56,197 vs. 59,405, *p* = 0.557), percentage of residents aged in 60 and older (17.83 vs. 18.21, *p* = 0.49), and total mortality between 2012 and 2013 (6.15 vs. 6.01‰, *p* = 0.52).

**Table 1:** **Characteristics of study sample in major cities in 2012 and 2013, China**.

	2012			2013			*p*-Value
	No. cities	Mean	SD	No. cities	Mean	SD	
**City-level daily data**							
Air pollution index (API)	120	65.05	26.41	120	75.99	41.81	<0.0001
Temperature (°C)	193	10.84	13.40	193	11.55	12.92	<0.0001
Heat index	193	46.30	26.41	193	47.66	25.40	<0.0001
Precipitation (0.1 mm)	193	21.98	82.96	193	21.05	85.54	0.039
Sunshine hours (h)	193	6.09	4.12	193	6.46	4.04	<0.0001
Pressure (kPa)	193	92.38	10.42	193	92.37	10.38	0.97
**City-level annual data**							
Population size (10,000)	120	564.95	425.16	120	568.98	430.18	
GDP per capita (Yuan)	120	56,197.63	29,178.59	120	58,405.30	28,971.20	0.557
% of residents aged ≥60	107	17.83	3.90	89	18.21	3.73	0.490
Total mortality (‰)	107	6.15	1.61	89	6.01	1.24	0.506

*No. cities, the number of cities with API reports; No. M, total number of API measures per year; GDP, gross domestic product*.

### Trend of API in 2012 and 2013

**Figure 1:**
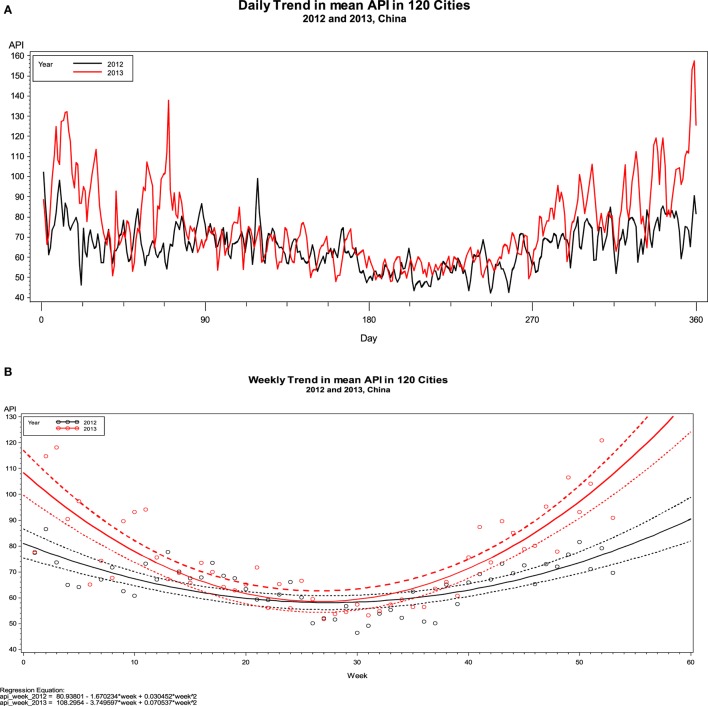
**Daily (A) and weekly (B) trend in air pollution index in 120 cities of China in 2012 and 2013, China**.

Figure 1A depicts the overall daily average APIs in 2012 vs. 2013. It shows API concentration was higher around the first 3 months (90 days), and the last 2 or 3 months of 2013 as compared with 2012. Figure 1B shows weekly API trend in 2012 vs. 2013. It is clear that mean APIs were lower in the months of July, August, and September in 2012 and 2013 (Figures 1A,B). By seasons, the highest average (±SD) of API values were observed in winter (72.47 ± 30.51), followed by spring (67.47 ± 26.13), fall (65.26 ± 23.86), and summer (55.16 ± 21.27) in 2012. A similar seasonal pattern was observed in 2013, except with a slightly higher API recorded in fall than that in spring. The corresponding average APIs in 2013 were 91.61 ± 48.09, 75.64 ± 35.18, 72.10 ± 34.54, and 57.54 ± 21.78 in winter, fall, spring, and summer, respectively. Table 2 shows that among the 120 cities, 5 cities (4%) in 2012 and 21 cities (18%) in 2013 had API >100 (defined as “slightly polluted” – a risk level for vulnerable population, including those who have pre-existing respiratory and/or heart disease, and children, and old adults) for ≥60 days in a year. Of the 120 cities, 16 (13%) in 2012 and 35 (29%) in 2013 experienced a maximum API >300 (defined as “severely polluted”/for all residents) over a year.

**Table 2:** **Number of days and cities with API more than 100, and maximum API in cities in 2012 and 2013**.

	2012		2013	
	No. cities	(%)	No. cities	(%)
**Days with API >100**				
0–14	39	32.50	64	53.33
15–29	33	27.50	12	10.00
30–44	28	23.33	13	10.83
45–59	15	12.50	10	8.33
≥60	5	4.17	21	17.50
Total	120	100	120	99.99
**Max API**				
≤100	9	7.50	10	8.33
101–200	80	66.67	61	50.83
201–300	15	12.50	14	11.67
>300	16	13.33	35	29.17
Total	120	100	120	100

### Spatial–Temporal Patterns of API

**Figure 2:**
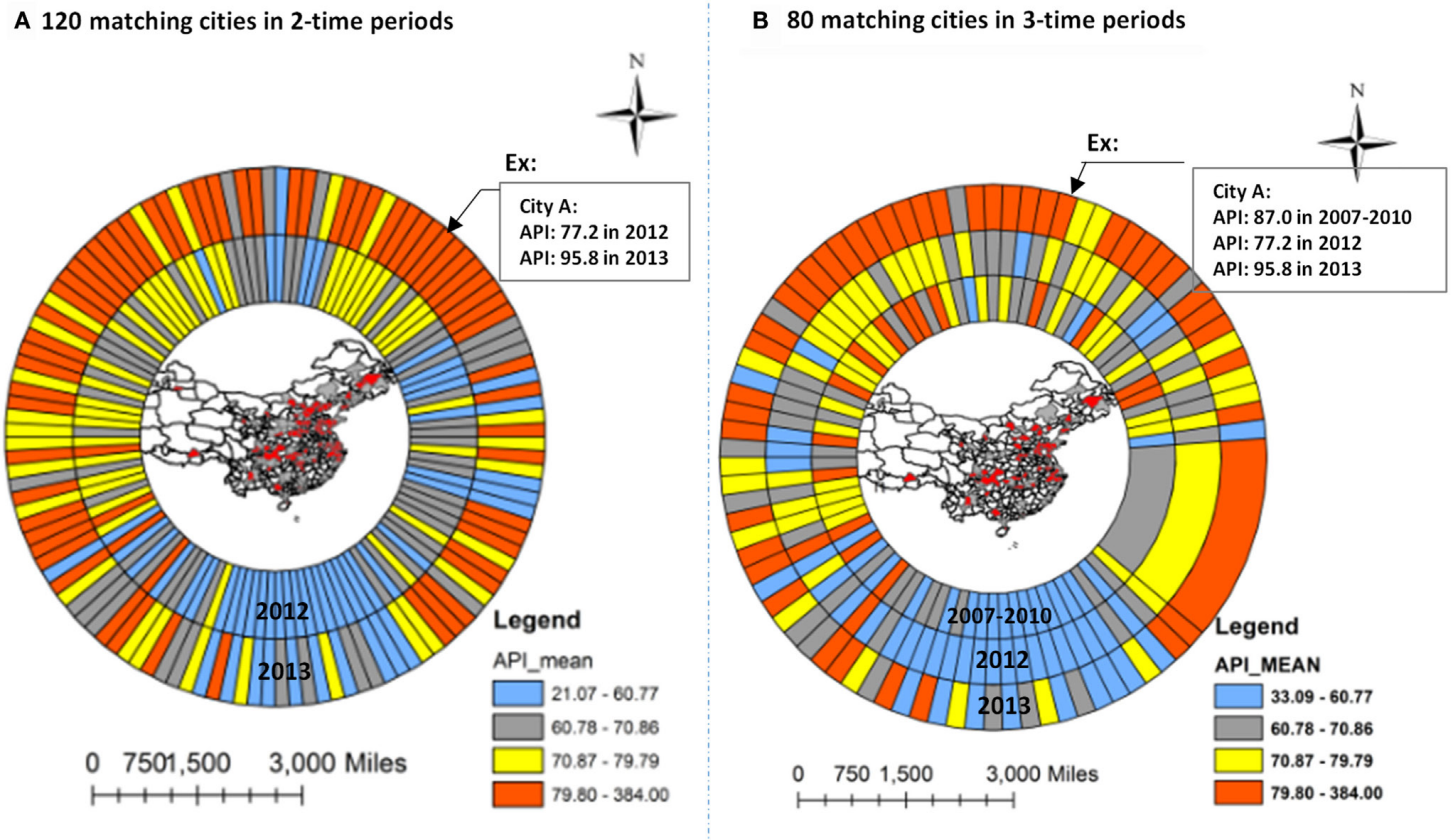
**GIS Ring Map analysis: trend in air pollution index in 120 cities of China by cities from 2012 to 2013 (A), and from 2007–2010 to 2013 (B)**.

Using GIS Ring Map technique, Figure 2 depicts significant variations in mean API across the 120 cities in 2012 and 2013, and the number of cities with an elevated API increased from 2012 to 2013 (Figure 2A, shown by different color), and from 2007–2010 to 2013 (Figure 2B). For example, in Figure 2A, the inside surrounding ring with 120 trapezoids represents mean API for each of the 120 cities in 2012, and the second (outside) surrounding ring with 120 trapezoids represents the corresponding cities with mean API in 2013. Four different colors represent the ranges of API by quantile, with blue representing the lowest API group (21.07–60.77), followed by gray (60.78–70.86), yellow (70.87–79.79), and red (≥79.80), respectively. In the study of 120 cities (Figure 2A), for example, City A had a mean API of 77.2 in 2012 (yellow) and 95.8 in 2013 (red). In the three time periods’ analysis (Figure 2B), 80 cities had mean API measures in 2007–2010, 2012, and 2013 (from inside, middle to outside rings). The annual average API in 2007–2010 was 87.0 in City A (red), it was 77.2 in 2012 (yellow), and 95.8 in 2013 (red). Of the 120 cities (Figure 2A), 73 (60.8%) experienced at least an average 10-unit increase in mean API from 2012 to 2013. Of the 80 cities in the three time periods’ study, 49 cities (61.3%) experienced a 10-unit increase in mean API from 2007–2010 to 2013.

### Correlation between API and Meteorological Factors

**Figure 3:**
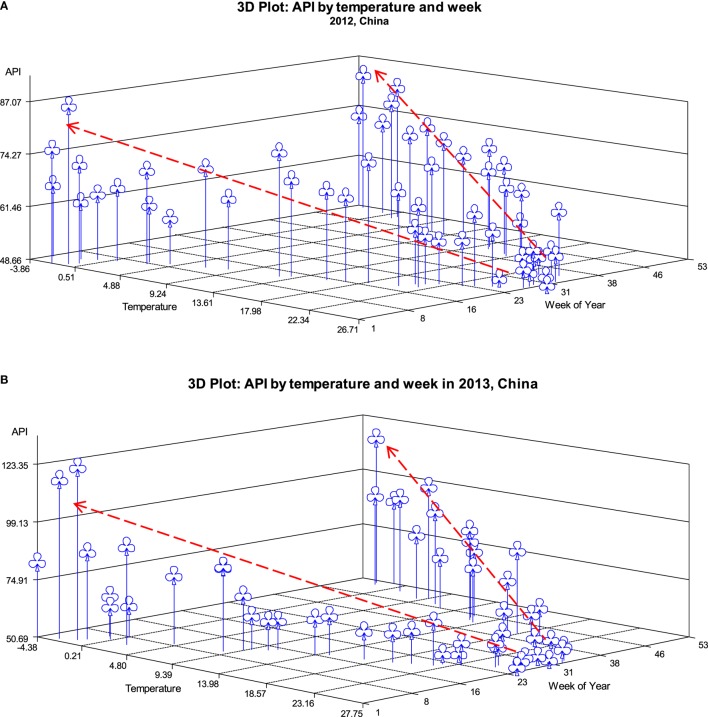
**Mean API by temperature and weeks in 2012 (A) and 2013 (B)**.

In the correlation analysis, cities with data for both API and meteorological measures (assessed by weekly averages) were included in 2012 and 2013. Among the matching cities (*n* = 49, with both API and climate factors measures), results from univariate correlation analysis indicated that for 2012 and 2013, a significantly negative correlation of average API with average temperature, HI, precipitation, and sunshine hours was observed by weeks, however, a significantly and positively correlation with pressure in 53 weeks of a year (top section in Table 3). A further analysis was conducted by stratification of seasons in order to examine whether the association between API and temperature still holds. The results show that this negative association of API with temperature and HI became non-significant in winter in 2012 and 2013. The values of the correlation coefficients of API with temperature and HI were higher in fall and summer in 2012, and in spring and fall in 2013 as compared with the other seasons. Figure 3 shows a three-dimensional relationship of API with time (53 weeks of a year) and temperature. It is clear, time and temperatures were significantly correlated with API, with a higher mean API in the early and late weeks of a year (i.e., around December, January, and February) and in cities with lower temperatures in 2012 (Figure 3A) and 2013 (Figure 3B).

**Table 3:** **Correlation between weekly average API and climate indicators in 2012 and 2013**.

	API in 2012			API in 2013					
	Week	*r*	*p*-Value	Week	*r*	*p*-Value
**By year**						
Temperature (°C)	53	−0.672	<0.0001	53	−0.767	<0.0001
Heat index	53	−0.670	<0.0001	53	−0.767	<0.0001
Precipitation (0.1 mm)	53	−0.720	<0.0001	53	−0.707	<0.0001
Sunshine hours (h)	53	−0.447	0.001	53	−0.548	<0.0001
Pressure (kPa)	53	0.648	<0.0001	53	0.696	<0.0001
**By season for TP and HI**						
Temperature (TP)						
Winter	12	−0.028	0.931	12	−0.301	0.342
Spring	14	−0.235	0.418	14	−0.688	0.007
Summer	13	−0.626	0.022	13	−0.352	0.239
Fall	14	−0.785	0.001	14	−0.657	0.011
Heat index (HI)						
Winter	12	−0.028	0.931	12	−0.301	0.342
Spring	14	−0.235	0.418	14	−0.688	0.007
Summer	13	−0.544	0.055	13	−0.374	0.208
Fall	14	−0.785	0.001	14	−0.657	0.011

To take into the consideration of the complex correlation between API, climates factors, and time (by weeks), results from panel analysis indicated that all meteorological factors were significantly correlated with API in panel pooled models (Table 4). Model 4 indicates that about 20% (*R*^2^, 20.8% for 2012, and 21.0% for 2013) of the variations in API could be explained by the differences in meteorological factors. Similar results were observed using panel one-way fixed time (weeks) effect models, except for a non-significant association between API and sunshine hours in 2012. Results of the panel fixed models suggest that for 2012 there was a variation of around 30% (*R*^2^, 28.5–33.0%), and for 2013 more than 40% of the variation in API could be explained by the differences in meteorological factors and time (weeks).

**Table 4:** **Panel analysis of the associations between daily API and climate factors**.

	Pooled model								One-way fixed effect model							
	1		2		3		4		1		2		3		4	
	Beta	*p*-Value	Beta	*p*-Value	Beta	*p*-Value	Beta	*p*-Value	Beta	*p*-Value	Beta	*p*-Value	Beta	*p*-Value	Beta	*p*-Value
**Year 2012**																
Sample size (*n*)	2597		2592		2575		2575		2597		2592		2575		2575	
Heat index	−0.005	<0.0001	−0.003	<0.0001	−0.003	<0.0001	−0.004	<0.0001	−0.321	<0.0001	−0.331	<0.0001	−0.353	<0.0001	−0.444	<0.0001
Precipitation (0.1 mm)			−0.002	<0.0001	−0.002	<0.0001	−0.002	<0.0001			−0.091	<0.0001	−0.102	<0.0001	−0.097	<0.0001
Sunshine hours (h)					0.000	0.906	0.004	0.033					−0.410	0.012	0.031	0.858
Pressure (kPa)							0.006	<0.0001							0.642	<0.0001
Fixed time effect (53 weeks)									Y		Y		Y		Y	
*R*^2^	0.134		0.202		0.197		0.208		0.285		0.314		0.312		0.330	
**Year 2013**																
Sample size (*n*)	1047		1047		1047		1047		2597		2592		2575		2575	
Heat index	−0.590	<0.0001	−0.510	<0.0001	−0.404	<0.0001	−0.423	<0.0001	−0.605	<0.0001	−0.650	<0.0001	−0.640	<0.0001	−0.659	<0.0001
Precipitation (0.1 mm)			−0.124	<0.0001	−0.183	<0.0001	−0.177	<0.0001			−0.105	<0.0001	−0.141	<0.0001	−0.133	<0.0001
Sunshine hours (h)					−2.936	<0.0001	−2.588	<0.0001					−1.701	0.0003	−1.418	0.003
Pressure (kPa)							0.630	0.009							1.114	0.0004
Fixed time effect (53 weeks)									Y		Y		Y		Y	
*R*^2^	0.145		0.165		0.205		0.210		0.402		0.413		0.421		0.428	

*Beta, regression coefficient*.

### Association between Annual Average API and Total Mortality Rates

Among 120 cities, we were able to collect reliable total mortality data from 107 cities in 2012 and 89 in 2013. Among the 107 cities, Shenzhen had the lowest total mortality rate (1.4/1,000 residents), and city of Yangzhou had the highest total mortality rate (10.2/residents) in 2012. Among the 89 cities, the city of Zhuhai had the lowest total mortality rate (2.4/1,000 population), and the highest total mortality rate (9.2/1000 population) was observed in the city of Fushun in 2013.

**Figure 4:**
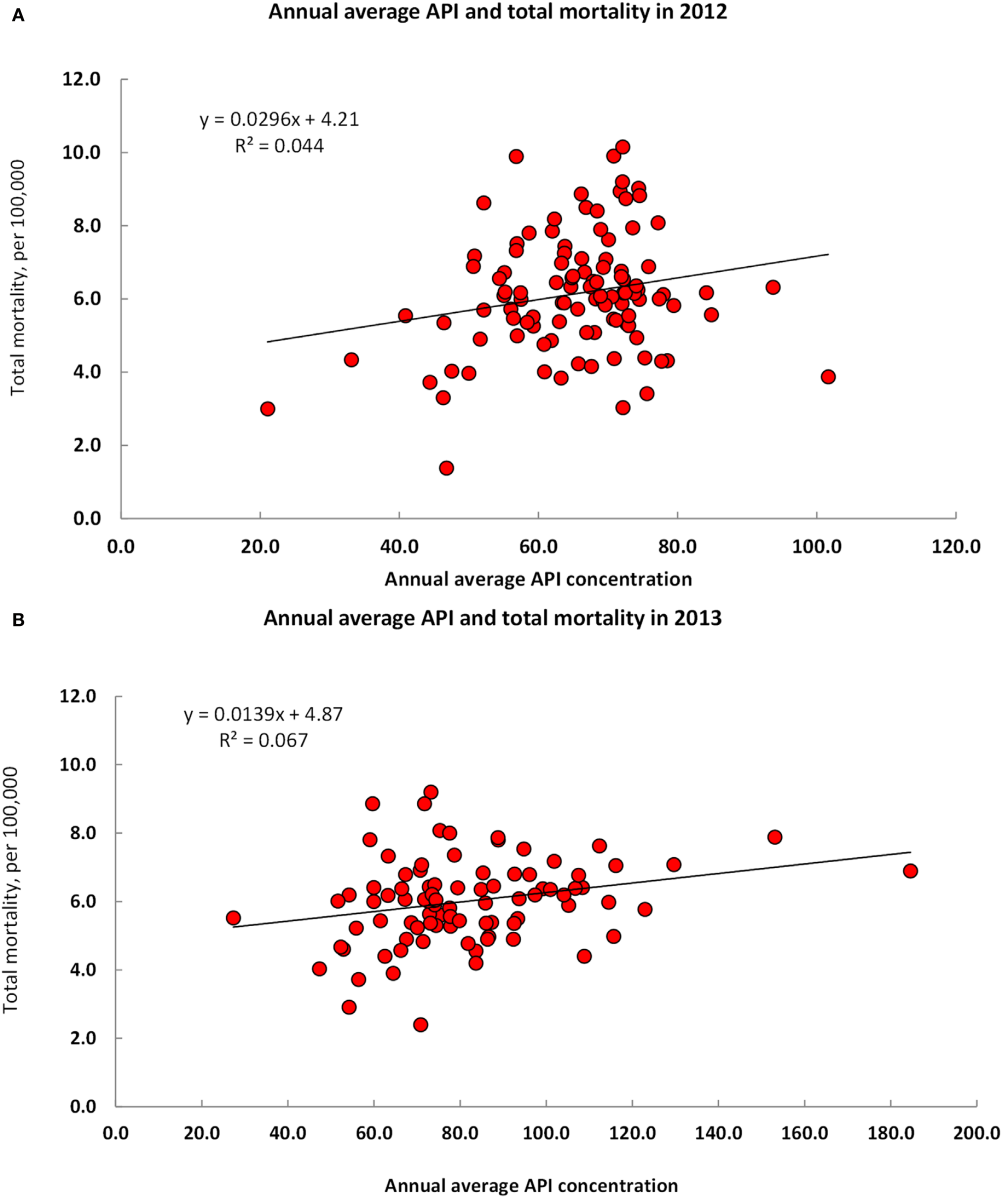
**Annual average air pollution index and total mortality rate in Urban Residents of China in 2012 (A) and 2013 (B)**.

Annual average APIs were positively and significantly correlated with total mortality rate in 2012 (*r* = 0.21, *R*^2^ = 4.4%, *p* = 0.030), and in 2013 (*r* = 0.26, *R*^2^ = 6.7%, *p* = 0.013) (Figures 4A,B). After adjustment for socioeconomic status (SES, assessed by GDP per capital), the association between API and total mortality remained statistically significant in 2012 (*r* = 0.21, *p* = 0.031) and in 2013 (*r* = 0.22, *p* = 0.039). An adjustment for the inclusion of both SES and the percent of residents aged 60 and older shows that the correlation coefficients of API with total mortality remained positive, although they became borderline significant in 2012 (*r* = 0.18, *p* = 0.062) and in 2013 (*r* = 0.21, *p* = 0.053).

Table 5 shows the results from sensitivity analysis. City-level air quality index measured in 2003 was positively associated with total mortality rate in 2012 (i.e., assuming a 10-year time-lag effect between exposure and health outcomes), although the correlation coefficient had borderline significance (*n* = 41, *r* = 0.304, *p* = 0.054). The same result was observed with a positive association between air quality index in 2004 and total mortality rate in 2013 (*n* = 38, *r* = 0.253, *p* = 0.125). A positive and significant association between air quality in 2008 and total mortality rates in 2012 (*n* = 76, *r* = 0.372, *p* = 0.001) was observed (i.e., assuming a 5-year time-lag effect between exposure and health outcomes). Significant associations between 4-year annual average air quality index in 2007–2010 and total mortality rates in 2012 (*n* = 76, *r* = 0.364, *p* = 0.001) and 2013 (*n* = 73, *r* = 0.261, *p* = 0.026) were observed (i.e., assuming a 5-year time-lag effect). On a possible 10-year time-lag effect, there was borderline significant association between a 4-year annual average air quality index in 2002–2005 and total mortality rates in 2013 (*n* = 71, *r* = 0.234, *p* = 0.049), but a positive and non-significant in 2012 (*n* = 74, *r* = 0.172, *p* = 0.143).

**Table 5:** **Correlation between air quality in early years and total mortality in 2012 and 2013**.

Air quality index	Total mortality					
	**2012**			**2013**		
	No.	*r*	*p*-Value	No.	*r*	*p*-Value
**Annual average**						
2003, 2004	41	0.304	0.054	38	0.253	0.125
2008, 2009	76	0.372	0.001	73	0.217	0.066
**4-year annual average**						
2002–2005	74	0.172	0.143	71	0.234	0.049
2007–2010	76	0.364	0.001	73	0.261	0.026

*No, no. of cities with available data for air quality measures and total mortality; r, correlation coefficient*.

*Annual average air quality index in 2003 and 2004 for testing a 10-year time-lag effect on total mortality in 2012 and 2013, and annual quality index in 2008 and 2009 for testing a 5-year time-lag effect, respectively*.

## Discussion

### The Main Findings

The main findings of the study highlight that (1) there was a significant increase in API from 2012 to 2013 in most cities of China. The highest average API was observed in winter, and the lowest in summer in the years 2012 and 2013. A significant spatial clustering of cities with high API was observed, with the highest API observed in Northwest China in 2012, and in North China in 2013. (2) Climatic factors remained significantly correlated with API after controlling time effect in both 2012 and 2013. (3) Increased API was positively and significantly associated with total mortality rate in univariate and partial (SES-adjusted) correlation analyses. The API – total mortality associations in 2012 and 2013 were further confirmed by testing early air quality measures in relation to total mortality in late years.

### Burden of Air Pollution in China

China is facing a great challenge of controlling air pollution, especially in urban cities and regions because of their differences in environmental protection and socioeconomic development. Chinese government has paid a great attention to the challenges since recent decade. National monitoring and report system for daily air quality has been established for major urban cities across the country (11). Findings from the study show that the average API significantly increased in most cities of the 120 reported cities from 2012 to 2013. A significantly higher proportion of cities that had an API >100 (defined as a “slightly polluted,” but being risk of disease and mortality for vulnerable populations) for ≥60 days in a year increased from 2012 to 2013 (4 vs. 18%, *p* < 0.001). The proportions of cities that had a maximum API >300 (defined as a “severely polluted,” being risk of disease and mortality for all populations) increased from 2012 to 2013 (13 vs. 29%, *p* < 0.001). The variations in API were significantly and spatially clustered across the country. Overall, North and Northwest China had higher API than the rest of the country. Although several studies examined the differences in air quality across local regions and cities, the present analysis is the first to use the newest released air quality data from 120 major cities and to test its spatial and temporal differences across the nation. Huang and his colleagues, using data from the city of Xi’an (located in the northwest of China), examined the trend of air quality (assessed by PM_2.5_) across 13 monitoring districts (stations) within the city. Their results showed that the mean PM_2.5_ concentration appeared decreased but no statistically significant change from 2013 to 2014 (*p* > 0.05). However, a significant clustering distribution of PM_2.5_ across the 13 districts were observed (51). Kai et al. evaluated air quality in Guangzhou (located in south central China), compared it with additional 11 large cities of China. They used air quality data monitored for a period of 6 years from 2000 to 2005, assessed by mean API and individual air pollutants of SO_2_, NO_2_, total suspended particular (TSP), PM10, and CO. Their findings suggested a significant variation in API and individual air pollutants among the 12 cities (10). Findings of our study add further new evidence using the largest dataset of 120 cities from the national surveillance system, and confirmed a significant increase in API in most cities from 2012 to 2013.

### API and Climate Change

Climate change can be caused in part by increased atmospheric concentrations of carbon dioxide and other green-house gases. It is likely to result in changes in temperature, humidity, amount, distribution, and intensity of precipitation events and bring about variation in intensity and frequency of certain extreme weather events (52). In our study, significantly negative associations of API with HI, precipitation, and positive association with atmospheric pressure were observed. A higher API in winter and lower value in summer has also been reported by other studies in China. For example, the concentration of PM_2.5_ was higher in winter and spring, but was lower in summer in previous studies conducted in the cities of Xi’an and Guangzhou of China (10, 51). We further confirmed the associations using multivariate panel analysis by taking into consideration of weekly changes in API during a given study year. A significant variation in air quality by seasons may be attributable to the natural climate changes, such as months that are cold less windy in winter, heavy rainfall in summer, and use of different types of heating sources. In winter, coal is the main source of heating in most cities of China, which may be attributed for a poor air quality, or/and for a combined effect of less windy, or inversion during cold months. A dry and dusty spring may account for a relatively higher concentration of air pollutants in spring. While in summer, there are lots of rainy and windy days, which can increase the sedimentation rate in air and increase diffusion of air pollutants. In fall, low temperatures and increased winds do not support diffusion of air pollutants (7, 51). Furthermore, it may be also due to the fact that industrialization in North China occurred much before that in South China. In general, cities in the North have energy consumption from coal and other sources to a greater extent than cities in South China, which may be partly attributable for higher concentrations of air pollutants in the North vs. the South. Although we are unable to examine the details of the associations between air pollutants, climate change, and energy use because of lack of the relevant datasets, findings from our study address the association of API concentration with climatic factors and seasonal variations. Of them, the seasonal variations in API may be partly explained by the different types of energy use (i.e., more coal used in winter for heating), and call for further research.

### Impact of Air Pollution on Urban Health

The association between air pollution and public health has been examined in several cities of China. These studies observed a positive association of air pollution with years of potential life lost, total mortality, and mortality from cardiovascular disease and respiratory diseases (3, 5–9, 15, 53). These early studies highlighted the importance of controlled air pollution in urban cities, although they had several limitations, including a small sample size and/or data gathered using different study designs and processes that lead a difficulty in conducting comparative studies across regions and times (7). Our study expanded previous studies by utilizing data from China national air and health surveillance systems through which we could successfully test and confirm a significant and positive association between API and total mortality. About 4–7% of the variations in total mortality can be explained by the differences in API across the study cities. The findings, thus, further address the serious impact of air quality on public health.

However, similar to other studies, this study has several limitations. First, data on air quality are analyzed for 120 cities that covers one-third of major cities in China. Therefore, findings from the study remain limited to its generalizability to the whole cities in China. Second, the associations of API with temperature, weekly and seasonal changes, and total mortality rates were analyzed cross-sectionally. Further analysis using prospective cohort data is needed to test the cause–effect association between exposure and outcomes. However, there is no reason to suspect that risk of mortality precedes high API. Furthermore, in the analysis of the time-lag effect between exposure and health outcomes, our results showed a positive correlation between mean API measured in 2007–2010 and mortality in 2012 and 2013. Several other studies have also demonstrated a significant association between air pollution and human health (54–56). Findings from our analyses are consistent with previous findings and support that there is a positive and significant correlation between air quality and total mortality (57). Third, we are unable to examine the association between individual pollutants and total mortality because data for the individual air pollutants are not available from China national report system. Nevertheless, because API is a standardized sum weighted indicator of five major air pollutants (particular matters, SO_2_, NO_2_, CO, and O_3_), and residents are not exposed to just a single air pollutant in the real world, API can be an efficient and valid indicator for the total burden of air pollution. Fourth, the associations between city-level API and health outcomes were analyzed using ecological analysis approach, a potential ecological fallacy may occur. Fifth, data for cities that had both measures of API and climatic factors were only available from 49 cities, this relative small sample size reduced the statistical power when we tested the association between API and mortality with adjustment for climatic factors (data not shown). The statistical powers were further reduced when we adjusted more covariates (SES, population density, and percentage of residents aged 60 and older). Certainly further studies with a large sample size are needed. Sixth, detail data on air quality and health outcomes remain limited from China. For example, city-level disease-specific mortality data and mortality rates by months and seasons are not publicly available. Therefore, we applied total mortality rate as the health outcome in this study. It is clear that further collaborative studies are needed to extend and confirm the current findings.

Despite these limitations discussed above, this study has several advantages as well. First, as compared with all previous published work, this study has the largest sample size on air quality measures from 120 major cities in 2012 and 2013, China. Second, the accuracy and quality of data sources, including API, meteorological factors, and total mortality are reliable as they applied standard study designs and data collection procedures supported by the Chinese government environmental and central healthcare organizations. The approach of using data from national surveillance systems offers a comparable and important baseline study for further studies. Third, the application of the spatial autocorrelation analysis technique in this study helps us to quantitatively test and confirm a spatial clustering of API across China. Fourth, to our knowledge, it is one of the first in the literature of the relevant studies; this study examined the changes in air quality using advanced GIS Ring Map technique. Fifth, a robust sensitivity analysis was conducted using historical air quality measures by taking into consideration a possible time-lag effect between exposures and health outcomes, which thus confirms a positive and strong association between air quality and total mortality.

In conclusion, using data from Chinese national surveillance systems on air quality, climatic factors, and health outcomes, findings of the study indicate that the burden of air pollution increased in the past years. Although API had significant variations across the country, it was found that API is significantly associated with climatic factors and with total mortality rates in major cities of China.

## Author Contributions

Drs. LL, CH, and MW contributed to the initial study design for the Drexel – SARI Co-Research and Education on Low Carbon and Healthy City Technology Study. Drs. SW, SM, and AF gave comments and support for the Drexel-SARI study. LL and XY contributed to the analysis design of this report, conducted the data analysis, and drafted the manuscript. HL participated in the data analysis. Drs. CH, MW, SW, SM, and AF gave important comments on the manuscript.

## Conflict of Interest Statement

The authors declare that the research was conducted in the absence of any commercial or financial relationships that could be construed as a potential conflict of interest.
