# Supplementary material for: Spatial–Temporal Analysis of Air Pollution, Climate Change, and Total Mortality in 120 Cities of China, 2012–2013
Source: Front Public Health. 2016 Jul 18;4:143. doi: 10.3389/fpubh.2016.00143 (PMC4947578; doi:10.3389/fpubh.2016.00143)
Supplement: Supplementary file 1 [file table_1.docx]

Supplementary table
